# Laying the Foundation for an Elementary School Sleep Education Program

**DOI:** 10.3390/children13010138

**Published:** 2026-01-18

**Authors:** Alzena Ilie, Peyton Williams, Gabrielle Rigney, Shelly K. Weiss, Sarah Bluden, Penny V. Corkum

**Affiliations:** 1 Department of Psychology and Neuroscience, Faculty of Science, Dalhousie University, Halifax, NS B3H 4R2, Canada; alzenailie@dal.ca (A.I.);; 2 Appleton Institute, School of Health Medical and Applied Sciences, Central Queensland University, Adelaide, SA 5034, Australia; 3 Department of Paediatrics, Faculty of Medicine, University of Toronto, Toronto, ON M5S 1A1, Canada; 4 School of Education, Monash University, Clayton, VIC 3800, Australia; 5 Department of Psychiatry, Faculty of Science, Dalhousie University, Halifax, NS B3H 4R2, Canada

**Keywords:** sleep, school-aged children, school-based program, needs assessment, sleep education, elementary school

## Abstract

**Background/Objectives:** Many elementary school-aged children (i.e., 5 to 12 years old) experience sleep difficulties that negatively impact their daytime functioning. Despite this high prevalence, sleep education is rarely included in school curricula and evidence-based interventions are limited. To better understand this gap, a needs assessment was conducted to inform the development of a sleep education program. **Method:** Semi-structured virtual interviews were conducted with 14 elementary school teachers in Nova Scotia, Canada. Participants were asked 20 questions about their students’ sleep and its impact, teachers’ needs and practices in sleep education, what a sleep education program would look like, and how it could be delivered. During the interview, participants watched the online *ABCs of SLEEPING* storybook as a potential foundation for developing a sleep education program, and interview themes were analyzed using deductive thematic analysis. **Results:** All teachers identified poor sleep as an issue impacting students’ behavior and learning, and reported that they had a lack of resources to teach sleep education. Teachers believed the storybook could be used with their students and integrated into the curriculum. Recommended modifications include making the storybook available for families, adding interactive activities and student discussions, providing teacher resources, and tailoring the content to be suitable for both lower and upper elementary school-aged students. Most teachers indicated that the storybook could be adapted for upper elementary students with more age-appropriate vocabulary and visuals. **Conclusions:** The findings from this needs assessment will inform the development of an elementary school sleep education program using the *ABCs of SLEEPING* storybook as the foundation of the program, while noting limitations such as sample diversity.

## 1. Introduction

Sleep is essential for health and well-being at all ages, especially for elementary school-aged children who are expected to learn, concentrate, and retain new information [1]. Approximately 30% of school-aged Canadian children (i.e., 5 to 12 years old) obtain less than the recommended 10 h of sleep per night, often having difficulties falling or staying asleep, and waking too early in the morning [2,3,4]. If these sleep difficulties reach clinical levels, they are diagnosed as insomnia, which is defined as frequent (three or more nights per week) and chronic (longer than three months) difficulties with sleep initiation and maintenance despite adequate opportunities [4,5]. Of the 30% of school-aged children with sleep difficulties, 10% have clinically diagnosed insomnia [6]. Both insomnia and insomnia symptoms negatively impact daytime functioning and impair skills needed for school success, including attention, memory, reasoning, and worsening behavioral and emotional problems [1,7]. These sleep problems are often due to poor sleep practices, including night-time screen use, social media, and unhealthy eating, as well as irregular sleep schedules, and poor sleep environments [7,8,9].

Over the past decade research has focused on developing and evaluating digital parent-implemented sleep interventions for their children [10,11,12]; however, interventions are often difficult to access, which results in many children not receiving treatment for their sleep problems [13,14]. Given these barriers, alternative strategies should be considered to effectively reach children in need of support. Schools have the ability to reach a large number of children and youth, providing an ideal setting to encourage children to adopt and maintain healthy lifestyles [15]. School programs targeting behavioral health outcomes (e.g., physical activity, dietary habits) have been found to improve both health and overall well-being [16]. Moreover, using the existing system can be an affordable and effective way to deliver health promotion programs [17]. Despite the critical role sleep has on daytime functioning and learning, sleep education is rarely included in school health curricula [18].

To date, research examining school-based sleep education programs has mostly been focused on high school students [18,19,20,21]. The *Healthy Sleep for Healthy Schools* *(HS4HS)* program was developed by Dr. Corkum, Dr. Rigney, and colleagues to be used with junior high and high school students with the goal of providing teachers with the knowledge and resources they require to teach students about healthy sleep. This program includes a teacher professional development component delivered online, in which teachers learn about the importance of sleep and healthy sleep practices. Once the teacher demonstrates mastery of the materials, teaching resources (e.g., PowerPoint slides, lesson plans, parent resources) are unlocked to be disseminated among their students. The program has been found to be useful, comprehensive, and easy to incorporate into school curricula [22,23]. To our knowledge, this is the only sleep education program developed and evaluated in Canada and Australia [22]. Preliminary evidence for effectiveness was demonstrated through positive reviews and improvements in sleep knowledge and practices following participation in the program [22]. During this research, educators indicated a need for a similar program for elementary school-aged students.

Despite the need for sleep education, few school-based sleep education programs for elementary school-aged students exist [18,20]. Evaluation of the six programs reported in the literature [17,24,25,26,27,28] indicated mixed results regarding the effectiveness of these programs. Though these studies show promising results, the findings are mixed regarding the effectiveness of these programs, often due to the lack of evidence-based design and the need for interventions to be developed and tailored for the educational systems they will be used in [24,28,29]. Most of the programs made for elementary schools were not specifically designed for the different grade levels, particularly lower elementary school-aged students (i.e., Grade Primary/Kindergarten to Grade 3) and upper elementary school-aged students (i.e., Grades 4 to 6), despite there being substantial differences in these grades in regard to cognitive functioning, executive functioning, and independence [30,31]. Additionally, educational policies and practices in Canada vary significantly across provinces and districts [15]. Therefore, the development of sleep education programs should be tailored to the specific characteristics of each education system [15].

Given the feedback from teachers in the Nova Scotia school system who requested an elementary school-based sleep education program, our research team decided to build a program to meet this need. As a first step, a needs assessment was deemed necessary. A needs assessment is critical in program development, as it identifies and prioritizes the needs of key stakeholders [32]. Gathering data from teachers directly ensures that the intervention is relevant and appropriately tailored to the context of the school environment [32]. Without a needs assessment, programs may fail to address the sleep-related needs of students within the school setting, thus leading to ineffective outcomes or irrelevant interventions [33].

The current research aimed to gather foundational information to inform the development of a sleep education program for teachers to deliver to elementary school-aged students. The needs assessment followed Witkin’s Three Phases of Needs Assessment, including (1) pre-assessment (exploration), (2) assessment (data gathering), (3) and post-assessment (utilization) [34]. The pre-assessment phase has been completed (e.g., informal discussion with educators in Nova Scotia, comprehensive literature review), while the current study focused on Phases 2 and 3, by conducting in-depth individual qualitative interviews with stakeholders (i.e., elementary school teachers) [33]. As a starting point in the development of an elementary school sleep education program, we explored the use of a children’s storybook, the *ABCs of Sleeping* [35], designed to help children learn about the importance of sleep and what healthy sleep practices include. It was originally written as a storybook to be read to children in Grades Primary/Kindergarten to Grade 3, but may be a potential foundation for a sleep education program for elementary school-aged students. The *ABCs of SLEEPING* storybook presents a mnemonic, created by Dr. Corkum and trainees, that encapsulates healthy sleep practices [36]. The mnemonic is: Age-appropriate Bedtimes and wake-times with Consistency, Schedules and routines, Location, Exercise and diet, no Electronics in bedroom or before bed, Positivity, Independence, and Needs met, equal Great sleep.

The research was guided by five key objectives: (1) to gather teachers’ perspectives on students’ sleep and its impacts on elementary school-aged students in the classroom and at school; (2) to determine the need for sleep education and learn what sleep information teachers are currently delivering in their classroom; (3) to determine if the *ABCs of SLEEPING* storybook can be used as the foundation for a sleep education program for the elementary school context, and if so, what additional supports/resources are needed, (4) to collect elementary school teachers’ recommendations for effectively delivering the *ABCs of SLEEPING* storybook in the classroom, and (5) to determine whether the above findings differ for lower elementary school-aged students (i.e., Primary to Grade 3) and upper elementary school-aged students (i.e., Grade 4 to Grade 6).

## 2. Method

### 2.1. Participant Selection

Eligibility included that the individual was currently working as an elementary school teacher (Primary to Grade 6) in Nova Scotia, Canada for at least one year and be fluent in English. The sample size was finalized at 14 participants when theme saturation was reached. This smaller sample size was expected as the study population is relatively homogenous, the research objectives narrowly defined, and the sample generated enough diversity of data [37,38].

### 2.2. Measures

Participant enrollment, consent, questionnaires, and interview scheduling were arranged through REDCap [39].

#### 2.2.1. Screening Questionnaire

This is an author-made, 5-item questionnaire that was used to verify that participants are eligible to participate in this study. The questionnaire includes items pertaining to the inclusion criteria of the study. The inclusion criteria are that teachers must teach at the elementary school level (Primary to Grade 6) in Nova Scotia, they must have worked as an elementary school teacher for at least 1 year, and they must be fluent in English. There were no exclusion criteria. The questionnaire took approximately 4 min to complete.

#### 2.2.2. Demographic Questionnaire

This is an author-made, 8-item questionnaire used to collect demographic information about the study sample, including the teachers’ age, gender, race/ethnicity, education level, the community where they teach, the grade they are currently teaching, and years of teaching experience. The demographic questionnaire was adapted from a questionnaire used in previous research in Corkum LABS [40]. The questionnaire took approximately eight minutes to complete. The data collected was used to describe the sample and determine the representativeness of the sample.

#### 2.2.3. Sleep Education Experiences (SEE) Questionnaire

This is an author-made, 6-item questionnaire used to collect information about teachers’ knowledge about sleep and experience teaching sleep information in the classroom. The questionnaire took approximately three minutes to complete. The data collected was used to describe the sample and their sleep knowledge before completing the interview.

#### 2.2.4. Qualitative Interviews

Virtual semi-structured interviews were held, with video and audio recorded and transcribed using the web-based Microsoft Teams Software (see Supplementary Table S1). The interviews were approximately 30 min long (*M* = 28.64; *SD* = 8.55; 18–55 min).

Researchers used a templated summary table, a researcher-created data collection form based on the methods used in previous qualitative studies [41,42]. The templated summary tables assisted in organizing qualitative interview data when note taking, coding, and when extracting themes during data analysis.

The 20 interview questions were divided into five sections. For the first set of questions (*n* = 3) participants were asked about their students’ sleep and the impact of sleep problems. For the second set of questions (*n* = 4) participants were asked about the need for sleep education and current practices in teaching sleep. Teachers then watched the online *ABCs of SLEEPING* storybook video, which required five minutes. Afterwards, the third set of questions (*n* = 5) was asked, which focused on how the *ABCs of SLEEPING* storybook could be used as the foundation of a sleep education program. The fourth set of questions (*n* = 7) focused on how the program could be delivered. Lastly, the fifth section provided participants with the opportunity to share any additional information they thought was important which had not been addressed in the interview.

### 2.3. Procedure

Recruitment was conducted through the investigators’ professional contacts within the education system in Nova Scotia and email invitations to teachers from the Accessible Strategies Supporting Inclusion for Students by Teachers (*ASSIST*) Study (Principal Investigator, P.C.) who had consented to being contacted for future studies coordinated in Corkum LABS.

Potential participants completed an initial screening questionnaire to confirm eligibility. To prevent fraudulent participation the screening questionnaire included a verification item asking what school the participant currently teaches at in Nova Scotia. Participants’ responses were cross-referenced with publicly available staff directories from Nova Scotia schools to confirm their names appeared on the listed faculty or staff; additionally, participants were required to provide a school-affiliated email addressed to authenticate participants’ status as a current educator within the province. Eligible participants were then automatically directed to an electronic Information and Consent form. Once consent had been provided, participants were automatically directed to complete the demographic questionnaire and questionnaire about their experiences related to teaching sleep. Teachers then provided their availability for the qualitative interview. Participants received reminder emails 24 h and two hours in advance of their interview.

Relevant information from the consent form was summarized during the semi-structured interview. After consent was confirmed, the researchers asked questions based on the interview guide and during the interview participants watched the online video of the *ABCs of SLEEPING* storybook. Throughout all interviews, two researchers were working together (PW, AI). One researcher conducted the interview, while the second researcher wrote summary notes of the participant’s answers and provided any support to the first researcher if necessary. To compensate participants for their time, participants who completed all study measures and interview were provided with a $30.00 CAD Amazon.ca online gift card.

### 2.4. Data Analysis

The demographic and SEE questionnaires were analyzed using SPSS Version 28 (IBM Canada). Descriptive statistics were calculated for the entire sample.

QSR International’s NVivo 12 analysis software [43] was used to organize qualitative data from interviews. Deductive Thematic Analysis [44] was applied to identify the key themes and program recommendations identified by participants. The User Experience Honeycomb framework developed by Morville & Sullenger [45] was used to assess seven important domains (see Supplementary Table S2 for a description of these domains) that impact the desire and motivation to use the *ABCs of Sleeping* storybook as the foundation of a sleep education program. Previous studies have shown that the User Experience Honeycomb framework provides a consistent and reliable method for evaluating program usability [40,46]. Once the interviews were completed, each researcher (P.W. and A.I.) independently coded the themes for each interview. The percentage of coding agreement between the coders was 95%. Discrepancies were resolved by discussing them with a senior member of the research team (P.C.).

Ongoing data analysis was conducted simultaneously with the qualitative interviews and data collection ceased upon data saturation of qualitative themes. Thematic saturation in the current study was defined as the point in data collection and analysis when the identification of new themes no longer contributed significant value or resulted in changes to the overall content analysis (i.e., all main themes of the study have already been found) [47]. In the current data analysis procedure, sub-themes had to be identified by three different participants (30%+) to be considered significant to ensure the themes were not isolated opinions but recurring patterns observed in several participants [48]. This approach captures a broader experience and perspective within the study population, enhancing the reliability and validity of the results [48].

## 3. Results

### 3.1. Sample Characteristics

Out of the 22 participants who consented to participate in the study, 14 completed the study by attending the virtual interview. Of the eight participants who did not participate in the interview, four withdrew due to their limited availability while the remaining four were automatically withdrawn due to a lack of response to follow-up emails.

The majority of participants identified as females (*n* = 13), while one participant identified as male. All participants identified as White. The majority of the participants (*n* = 9) resided in towns or rural areas, while the rest (*n* = 5) lived in a city. Participants had an average age of 40 years old (*M* = 40.35; *SD* = 10.94; 26–59 years). Half of the participants (*n* = 7) taught at the lower elementary level (i.e., Primary to Grade 3), and half (*n* = 7) taught at the upper elementary level (i.e., Grade 4 to 6). Further demographic information regarding study participants is in Table 1.

In terms of the teachers’ experiences in sleep education, four teachers reported they had ‘none’ to ‘not very much’ knowledge regarding the concept of sleep education and its goals, while ten teachers reported they were ‘somewhat’ to ‘very knowledgeable’. Most teachers (*n* = 9) reported having never taught sleep information in their curriculum, while five teachers reported that they had. All teachers agreed that sleep education is needed. Further information regarding experiences in sleep education is in Table 2.

### 3.2. Teachers’ Perspectives on Students’ Sleep and Its Impacts

At the beginning of the interview, teachers were asked questions about their students’ sleep. Responses are presented descriptively rather than thematically. The majority of teachers (*n* = 12) reported that their students were not aware of the importance of sleep. In terms of sleep problems, teachers reported delayed bedtimes (*n* = 12), falling asleep in class (*n* = 7), difficulties falling asleep (*n* = 5), daytime sleepiness (*n* = 3), and early morning awakenings (*n* = 2) in their students. Teachers also reported that, on average, 31% of students do not sleep well.

When asked about the impact of poor sleep, five themes were identified from the data (see Supplementary Table S3 for quotes). The most prevalent theme identified (*n* = 13) was student tiredness and low energy. Teachers (*n* = 12) noted negative impacts on their students’ mood and behavior (e.g., irritability, emotion regulation, externalizing behaviors), and had difficulties completing schoolwork/tasks when they did not sleep well. Teachers reported reduced focus and attention (*n* = 8), and learning difficulties among students (*n* = 7).

### 3.3. Teachers’ Needs and Current Practices in Sleep Education

When asked about their needs and current practices in sleep education, five themes arose from the data (see Supplementary Table S4). The majority of teachers (*n* = 13) recognized the need for sleep education; however, many teachers (*n* = 12) identified a lack of accessible evidence-based resources to effectively deliver sleep information. Most teachers (*n* = 12) reported engaging in informal conversations with students while nine used strategies to help students manage tiredness (e.g., mindfulness, yoga, movement). Of note, many teachers (*n* = 9) emphasized the need to reinforce sleep education at home.

### 3.4. Teachers’ Thoughts on Program Development

Teachers’ feedback on the potential use of the *ABCS of SLEEPING* storybook as a foundation for a sleep education program for elementary school-aged children were organized using the relevant domains of the User Experience Honeycomb framework (Morville & Sullenger, 2010). Their comments were grouped into 16 themes within the five relevant domains: (1) program credibility (one theme), (2) program accessibility (two themes), (3) program desirability (three themes), (4) program valuableness (three themes), and (5) program usefulness (seven themes) (see Supplementary Table S5 for quotes).

All teachers expressed high confidence in the storybook, likely as it is evidence-based and written by clinical sleep researchers. For program accessibility, most teachers (*n* = 10) emphasized improving accessibility for families (e.g., sending the book home, handouts for parents), and six teachers recommended translating the storybook for non-English-speaking families. All teachers (*n* = 14) found the storybook to be visually and thematically appealing. Half of the teachers (*n* = 7) offered suggestions for the illustrations, such as different forms of screen usage and more detailed images. Some teachers (*n* = 3) also mentioned the need for more diversity in the characters.

When teachers were asked whether the information and content in the storybook would be relevant and helpful for their students, all teachers reported that the impact of electronics on sleep provided valuable information, as most of the teachers (*n* = 9) identified electronics as the key cause of sleep problems in their students. Half of the teachers (*n* = 7) reported the content regarding sleeping in the same environment and having a bedtime routine was valuable and relevant, and six teachers appreciated the content on nutrition and exercise. All teachers recommended adding discussion points to facilitate discussions in the classroom about the storybook content. Most teachers (*n* = 8) recommended incorporating an activity book (i.e., collection of activities including coloring pages, writing activities); some teachers (*n* = 5) felt activity books may not be helpful, as one may find it overwhelming to have every activity all at once. Furthermore, ten teachers recommended incorporating parent involvement to support and reinforce their child’s sleep practices at home.

Teachers provided further suggestions for program usefulness, including adding individual student activities (*n* = 10), such as goal-setting activities or engaging in an activity for each letter of the *ABCs of Sleeping* mnemonic. Increasing socially sensitive content was emphasized by some teachers (*n* = 6), pointing out there are some situations that are not addressed, especially for children from lower socioeconomic status (e.g., not having their own bedroom at home, lacking a quiet space to sleep; see Supplementary Table S5).

### 3.5. Teachers’ Recommendations for Program Delivery

When teachers were asked about their thoughts on program delivery, eight themes surfaced and were organized by the remaining two relevant domains of the User Experience Honeycomb framework (Morville & Sullenger, 2010): (1) program findability (two themes) and (2) program usability (six themes) (see Supplementary Table S6 for quotes). In terms of program findability, most teachers (*n* = 13) reported that a physical copy of the storybook offered advantages (e.g., planning purposes, limiting screen time). Conversely, ten teachers reported the advantages of having a digital version (e.g., accessing through the classroom website, projecting for group viewing). In terms of the program’s usability, all teachers believed the program could be effectively integrated and delivered within their current curriculum. When teachers were asked how they would discover a sleep education program, all teachers noted a variety of methods, including through school administration, newsletters, conferences, and colleagues.

The majority of teachers (*n* = 13) reported needing additional resources and guidance to teach about sleep with the storybook, such as an online learning module, and supplemental teaching resources (e.g., lesson plans, slideshows). A few teachers (*n* = 5) felt that no additional resources were needed if the program only involved reading the storybook. The majority of teachers (*n* = 12) suggested varying frequencies for how often they would use the storybook (e.g., twice to three times, every year, daily). Furthermore, some teachers (*n* = 8) recommended times for incorporating the storybook into the classroom, such as during snack time, integrating it into students’ daily schedules, and during silent reading (see Supplementary Table S6).

### 3.6. Teachers’ Needs Based on Lower and Upper Elementary School-Aged Students

When the qualitative data was compared between lower elementary and upper elementary, three themes were identified from the data (See Supplementary Table S7 for quotes). When asked about what grade level for which the storybook would be most appropriate, nine teachers reported it would be most suitable for lower elementary and five teachers reported it would be most suitable for both upper and lower elementary. In terms of the vocabulary level, nine teachers reported the vocabulary is at an appropriate level for their students; two lower elementary teachers reported that it may be too complex and two upper elementary teachers reported it may be too simple. When asked about the age level of the content, six lower elementary teachers and six upper elementary teachers felt it was at an age-appropriate level; however, twelve teachers provided suggestions for presenting the content in a more mature way for upper elementary school-aged students (e.g., illustrations of older students, mature visuals).

## 4. Discussion

The purpose of the study was to gather foundational insights to guide the development of a sleep education program for elementary school teachers and their students. The researchers collected information through questionnaires and semi-structured interviews to explore teachers’ perspectives on their students’ sleep, the impact of poor sleep in the classroom/school, current practices in sleep education, what a sleep education program would look like, and how it could be delivered in the classroom. Additionally, teachers reviewed the ABCs of SLEEPING storybook and provided feedback on its potential to be used as the foundation of the sleep education program. The findings showed that all teachers recognized poor sleep as a major factor impacting students’ success at school. They all highlighted the need for sleep education but noted a lack of available resources. Teachers found the storybook accessible, credible, desirable, valuable, useful, findable, and usable. Some teachers suggested changes for improvement, including making the storybook accessible to families, adding interactive activities and discussions for students, and providing additional teaching resources for effective delivery.

The first research objective aimed to determine teachers’ perspectives on students’ sleep and its impacts. All teachers identified poor sleep negatively impacting daily functioning at school, including students’ energy levels, mood and behavior, learning, schoolwork completion, and focus and attention. Our findings are consistent with previous research, which links sleep problems to greater levels of student conflict, increased inattention, academic difficulties, externalizing symptoms, and more general behavior problems [49,50]. The rates of sleep problems reported by teachers in this study (~31% of students not sleeping well) were representative of the national average of ~30% of Canadian parents reporting sleep problems in their school-aged children [3,4]. Some teachers indicated that as many as 65% of their students do not sleep well. A potential factor to explain this high prevalence of sleep problems could be the increasing use of technology during the evening among children, which is associated with shorter sleep duration and delayed sleep onset [51]. This is consistent with teachers’ reports in the current study, which identified the use of electronics as one of the main causes of sleep problems.

The second research objective aimed to determine the need for sleep education, and to learn what teachers are currently doing related to teaching about sleep. All teachers in the current study believed there is a need for sleep education though also noted the challenges in providing such due to the lack of available resources. Gruber and colleagues [15] found that teachers often do not have the opportunity to acquire information on healthy sleep practices or deliver that information to their students due to time constraints. Furthermore, most teachers have not been trained about the importance of sleep or strategies that can help improve sleep [15]. Additionally, the majority of teachers (n=12) reported that informal conversations were their primary method to address sleep issues; informal conversations can build awareness and influence students’ perspectives on healthy sleep behaviors though they may not lead to changes in sleep behavior [52]. This contrasts with evidence-based sleep programs, which have been found to change sleep knowledge and behaviors [53,54]. Furthermore, teachers reported the need to reinforce sleep education at home. Research highlights the need for school-based interventions to be supported through a collaborative approach that includes family involvement [21]. Two studies that encouraged parental involvement demonstrated that the effectiveness of sleep interventions increased when parents were involved [17,55]. This supports the notion that incorporating both teacher and parental involvement in sleep education can enhance the effectiveness of such programs.

The third research objective aimed to determine if the *ABCs of SLEEPING* storybook could be used as the foundation for a sleep education program in the elementary school context. All of the teachers found the storybook to be visually appealing and desirable, with the information provided in the storybook presented in a way that contributes positively to the user experience. For example, the teachers interviewed praised the messages in the storybook, as well as the diversity, format, colors, lettering, and illustrations used. Previous research supports the effectiveness of using the *ABCS of SLEEPING* mnemonic with parents to promote healthy sleep practices in their children [10]

Some teachers offered suggestions for improving the *ABCs of SLEEPING* storybook, particularly regarding the illustrations, culturally sensitive information, and diversity of the characters. Teachers noted that they teach a significant number of students from poorer socioeconomic and minority backgrounds and as such programs need to be more culturally sensitive. According to Sosso & Khoury [56], children from lower socioeconomic backgrounds are more likely to experience sleep difficulties than their higher-income peers. These insights suggest that developing educational materials which represent the diverse backgrounds and experiences in a specific regional area may make a sleep education program more applicable and relevant.

Furthermore, to support effective implementation of the program, teachers emphasized the need for additional teaching resources and guidance to effectively teach sleep education using the storybook. Findings from the *HS4HS* program designed for junior high/high school showed that incorporating teaching materials and resources made the program more usable and facilitated program implementation [22]. This suggests that similar resources would be valuable and helpful for a sleep education program for elementary school-aged students as well.

The fourth research objective aimed to determine the recommendations elementary school teachers have for delivering the *ABCs of SLEEPING* storybook. Teachers expressed a preference for both physical and digital versions of the storybook, noting different advantages in each format. While there is little evidence suggesting one way of delivery is better than the other in changing sleep behavior, student engagement and improved sleep outcomes likely depend on the teacher’s ability to deliver the program effectively [20]. Our study’s findings support this, as teachers expressed a preference for a combination of both formats to enhance student engagement and accessibility.

The fifth and final research objective aimed to determine whether the findings from the research differed based on lower elementary versus upper elementary. Most teachers identified that the storybook was most suitable for lower elementary school-aged students and could be adapted for upper elementary school-aged students with adjustments to vocabulary and content. Teachers suggested including illustrations of older students, more mature visuals, and content that resonates with the needs of upper elementary students. As discussed previously, there are significant differences in cognitive and executive functioning between these grades [30,31]. Tailoring a school-based sleep education program to the developmental needs of school-aged children, such as addressing varying levels of autonomy and social stimulation, can increase the effectiveness of the program in promoting changes in sleep behavior [21].

### 4.1. Strengths

The current study has several strengths, such as its use of theoretical frameworks, which guided and structured the formulation of research questions and the organization of the research data. By adopting a user-centered design approach, following Witkin’s Three Phases of Needs Assessment [34], the study ensured that the development of the sleep education program would be effective and useful for teachers. This approach underscores the study’s focus on creating an effective intervention that is based on a clear understanding of the specific needs of key stakeholders [34]. Additionally, the use of the User Experience Honeycomb framework [45] is a notable strength, as it assesses seven essential domains of usability. By evaluating these domains, the study ensures that the *ABCs of SLEEPING* storybook meets the needs and preferences of its users, which will enhance the program’s potential for successful engagement and retention [45]. By only interviewing lower and upper elementary school teachers, the researchers were able to focus on the needs of the current Nova Scotia elementary school system and gain knowledge to help inform the development of a sleep education program. This distinction is important, as despite being grouped together within elementary education, lower and upper elementary school-aged students exhibit developmental differences, and therefore should be considered separately [30,31]. As such, the findings of this research may have important implications for the adaption or implementation of similar sleep education programs across Canada in other educational systems.

### 4.2. Limitations

This study is not without limitations. First, it is likely that only teachers who believed there was a need for sleep education participated in the study, as reported in the SEE questionnaire. This introduces a potential bias as those who see sleep education as necessary may be more inclined to support the development of a school-based sleep education program. Additionally, as all participants identified as White, and the recruitment of participants was carried out relying on investigators’ professional contacts and previous research participants, the current sample may not be representative of a more diverse group of teachers in Nova Scotia. Teachers that are racially diverse may have different perspectives about sleep or sleep education in their students. Lastly, the current study used the approach of requiring themes to be identified by at least three different participants (30%+) to be considered significant. This was done to ensure the identified themes represented recurring patterns observed across multiple participants to capture a broader, shared experience and perspective within the study population [42,48]. However, this approach introduces a potential bias, as it may overlook valuable insights, different perspectives, and experiences that were not shared by a larger portion of the sample [48].

### 4.3. Implications and Future Research

The first step in advancing this research will be to use the findings from this study, in conjunction with the findings from the Australian study, to develop the *HS4HS Elementary* sleep education program. The Australian study involved focus groups with 15 elementary school teachers who were asked to review and provide feedback on the *HS4HS* for junior high/high school program in order to meet the needs of elementary school teachers. The research will use these findings combined with existing clinical and research knowledge, to develop an elementary school sleep education program. Following this, the program will be tested for its usability and its effectiveness in improving sleep outcomes among elementary school-aged students in Canada and in Australia.

In summary, the findings of this study underscore the critical need for sleep education programs in elementary schools in Canada. Teachers recognize the negative impact of poor sleep on students’ academic and behavioral outcomes and expressed the need for evidence-based resources to address this issue. The *ABCs of SLEEPING* storybook, with some modifications, was thought to provide a strong foundation for a sleep education program for elementary school-aged students. It is hoped that this research will help to inform the development of a sleep education program for elementary school teachers in order to improve sleep knowledge, sleep quality, and overall well-being in elementary school-aged students.

## Figures and Tables

**Table 1:** Teacher Demographic Variables.

Demographic Details	Total (*N* = 14; 100%)
**Teacher Gender**	
Female	13 (92.9%)
Male	1 (7.1%)
**Teacher Ethnicity**	
White	14 (100%)
**Community**	
Rural	5 (35.7%)
Town	4 (28.6%)
City under 500,000 people	4 (28.6%)
City over 500,000 people	1 (7.1%)
**Highest Education**	
Bachelors/Undergraduate Degree or Teacher’s College (e.g., B.A., B.SC., B.Ed.)	7 (50%)
Master’s Degree (e.g., M.A., M.Sc., M.Ed.)	7 (50%)
**Current Grade Teaching**	
*Lower Elementary* Grade 1	1 (7.1%)
Grade 2	1 (7.1%)
Grade 3	3 (21.4%)
*Upper Elementary* Grade 4	0 (0%)
Grade 5	1 (7.1%)
Grade 6	3 (21.4%)
Other (i.e., Split Class)	5 (35.7%) (2 Lower/3 Upper)
**What Grade Participant has Taught in Teaching Career**	
Elementary (Grades 1–6)	14 (100%)
Junior High School (Grades 7–9)	5 (35.7%)
Senior High School (Grades 10–12)	4 (28.6%)
**Demographic Details**	**M** ** (** ***SD)***
Teacher Age	40.35 (10.94)
Length of Time Teaching	14.64 (9.89)
Number of years Taught in Elementary	13.35 (8.39)

**Table 2:** Teacher Experiences in Sleep Education.

Sleep Education Experiences	Total (*N* = 14; 100%)
**Teacher Knowledge Level**	
No knowledge of this subject	1 (7.1%)
Not very knowledgeable	3 (21.4%)
Somewhat knowledgeable	8 (57.1%)
Knowledgeable	1 (7.1%)
Very knowledgeable	1 (7.1%)
**Experience Teaching Sleep**	
Never Taught About Sleep in Curriculum	9 (64.3%)
Taught About Sleep in Curriculum	5 (35.7%)
**Sources of Information Accessed on Sleep Education**	
No Information Accessed	10 (71.4%)
Accessed Sleep Information	4 (28.6%)
**Types of Resources Accessed**	
Readings Materials on Sleep Education	4 (28.6%)
Sleep Education Courses	1 (7.1%)
Sleep Education Workshops	1 (7.1%)
Other	1 (7.1%)
**Belief in the Need for Sleep Education in Curriculum**	
Strongly agree	8 (57.1%)
Agree	6 (49.2%)
Neutral	0 (0%)
Disagree	0 (0%)
Strong Disagree	0 (0%)
**Comfort Level with Teaching Sleep Education**	
Strongly agree	1 (7.1%)
Agree	6 (42.9%)
Neutral	3 (21.4%)
Disagree	4 (28.6%)
Strongly Disagree	0 (0%)
**Teacher Previously Used or Reviewed a Sleep Education Program**	
Yes	0 (0%)
No	14 (100%)

## Data Availability

The datasets generated and analyzed during the current study are not publicly available due to keeping participant’s identity and information confidential. The researchers do not have ethical approval to share the data.

## Supplementary Materials

The following supporting information can be downloaded at: https://www.mdpi.com/article/10.3390/children13010138/s1, Supplementary Table S1. *HS4HS* Needs Assessment Qualitative Interview Guide; Supplementary Table S2. User Experience Honeycomb Framework Domain Definitions; Supplementary Table S3. Teachers’ Perspectives on the Impacts of Poor Sleep on their Students (Themes); Supplementary Table S4. Teachers’ Needs and Current Practices in Sleep Education (Themes); Supplementary Table S5. Teachers’ Thoughts on Program Development (Themes Organized by User Experience Domains); Supplementary Table S6. Teachers’ Thoughts on Program Delivery (Themes organized by User Experience Domains); Supplementary Table S7. Teachers’ Needs Based on Lower and Upper Elementary Students Themes.
