# Supplementary material for: Laying the Foundation for an Elementary School Sleep Education Program
Source: Children (Basel). 2026 Jan 18;13(1):138. doi: 10.3390/children13010138 (PMC12839998; doi:10.3390/children13010138)
Supplement: Supplementary file 1 [file children-13-00138-s001.zip › children-4039956-supplementary.pdf]

Supplementary Table S1. ***HS4HS*** **Needs Assessment Qualitative Interview Guide**

Purpose	Question
**Element 1: Sleep and Its Impact on School-Aged Children**	**I am going to begin this interview by asking you three questions to learn more about your student's sleep and how it impacts them in your classroom and at school.**<br><br>1. Do you think your students are sleeping well? Do they ever talk about being tired?<br><br>2. Do you think some of your students are having sleep problems?<br>- **Prompt:** If so, what percentage of students in your class seem to have sleep problems and/or are tired during the day?<br>- **Prompt:** Do these sleep problems have an impact on your classroom?<br>- **Prompt:** How do sleep problems impact your students' functioning at school?<br><br>3. Do you think your students understand the importance of sleep and how it impacts their functioning during the day?
**Element 2: Needs and Current Practices in Sleep Education**	**Now we are going to change our attention to the next four questions focusing on needs and current practices in sleep education.**<br><br>4. Do you think there is a need for sleep education for students in schools?<br><br>5. Do you talk to your students about sleep?<br>- **Prompt (If yes):** What do you tell your students about sleep? Do you use any resources when teaching them about sleep?<br>- **Prompt (If no):** Do you think it's important to talk about sleep with your students?<br><br>6. Do you know if other teachers are providing any sleep education to their students in their classrooms?<br>- **Prompt (If yes):** Do you know if they are using specific programs or resources to deliver this information to their students?<br><br>7. What would you like to see in a sleep education program?
**Element 3: Program Development**	**Now, I am going to show you the *ABCs of SLEEPING* storybook video. Then, I am now going to ask you five questions to learn more about your thoughts on the storybook, if it would meet your needs and be a helpful resource as a school teacher.**

	(Show the *ABCs of SLEEPING* storybook video – the interviewer will share their screen on the Microsoft Teams call to do this)<br><br>8. What were your first impressions of the online book?<br><br>9. Did you find the information relevant to your students?<br>- **Prompt:** Why is it relevant/why isn't it relevant?<br><br>10. Do you think the book is at an appropriate learning level for your classroom?<br>- **Prompt (If no):** Do you think the information needs to be presented at a higher or lower level?<br>- **Prompt:** What age range do you think the ABCs of SLEEPING storybook is appropriate for?<br><br>11. Do you think the book would be a helpful resource in your classroom to teach children about sleep?<br>- **Prompt:** Why would it be helpful/why would it not be helpful?<br><br>12. Do you have any other ideas about how to make the ABCs of SLEEPING storybook interesting and relevant to school-aged children?
**Element 4: Program Delivery**	**We are now on to our last set of seven questions about the delivery of the sleep education program.**<br><br>13. Do you see any challenges with using the online book in the classroom or with the content in the book? Would a hard copy be better?<br><br>14. Does the fact that this book is evidence-based and was written by clinical researchers with expertise in sleep factor into your decision to use a resource like this in the classroom?<br>- **Prompt:** How does it influence your decision?<br><br>15. How would you use this book in your classroom?<br>- **Prompt:** How often would you read the book in your classroom?<br>- **Prompt:** Would you send the book home with students if possible?<br><br>16. Do you think you need additional support/resources for your own knowledge to teach the ABCs of SLEEPING storybook in your classroom?

	- **Prompt**: Would an online learning module with the background sleep knowledge be useful?<br><br>17. Would you need any additional resources to teach about healthy sleep using the book?<br>- **Prompt:** Would a document with "Discussion points" to talk about in the classroom to go along with the book be helpful for you?<br>- **Prompt:** How about an "activity book" for students to complete independently?<br><br>18. Do you think a sleep program that incorporated the ABCs of SLEEPING storybook would fit in with your other curriculum for health studies?<br>-**Prompt:** How might it have to be adapted?<br><br>19. At your school, how would you find out about an online program to help teach about healthy sleep?
**Closing**	20. In closing, I wanted to ask if there is anything we haven't touched on yet that you would like to mention?

Supplementary Table S2. User Experience Honeycomb Framework Domain Definitions

**Usefulness**	A helpful resource
**Usable**	User friendly; can be navigated with ease
**Findable**	Desired information can be located
**Desirable**	Visually appealing, presented in such a way that contributes positively to user experience
**Valuable**	Information provided is valued by the user
**Accessible**	An average user would feel comfortable navigating the program and be able to understand the information presented
**Credible**	Information is perceived as coming from a credible authority, the user trusts the information presented

Note: Based on Morville and Sullenger's 2010 User Experience Honeycomb Framework. These seven domains interact with each other which affects user experience.

Supplementary Table S3. Teachers' Perspectives on the Impacts of Poor Sleep on their Students (Themes)

Tiredness and Low Energy	***13 (92.9%)***
"Students say they didn't sleep well, or they didn't sleep long enough, or they didn't sleep at all." (Grade 6 Teacher)<br>"A decent chunk of students are complaining, or I'm noticing that they're too tired to be at the right level, that they should be for the day" (Grade 1 Teacher)	
Mood & Behaviour Challenges	***12 (85.7%)***
"They'll be more irritable during the day and with different interactions that they have with other people less patient." (Grade 6 Teacher)<br>"They're quicker to react to problems than they normally wouldn't be. Small problems become big problems." (Grade 1 Teacher)	
School Work/Task Completion	***12 (85.7%)***
"I find that when they're more tired, then it's more difficult to actually like start on the hard stuff. If we're doing math, for example, and trying to get through a new unit. It's difficult if they're not willing to learn, so that can be challenging." (Grade 4/5 Teacher)<br>"It also impacts whether or not they do homework or they get their other work done as far as finishing assignments, a lot of ones who are tired a lot, don't finish their work." (Grade 6 Teacher)	
Reduced Focus and Attention	***8 (57.1%)***
"Poor sleep impacts their ability to focus and pay attention; it makes it very difficult for them." (Grade 2 Teacher)<br>"Definitely sleep problems have an impact. If they're too tired to pay attention. Their brains aren't as focused during class" (Grade 4/5 Teacher)	
Learning Difficulties	***7 (50%)***
"They're not able to do the same things that their peers are because they are not able to take in the information, and it's impacting their learning." (Grade 6 Teacher)<br>"They don't learn as well as the other kids. Like you can see a difference. The kids who have a good sleep pattern just learn better." (Grade 3 Teacher)	

Supplementary Table S4. Teachers' Needs and Current Practices in Sleep Education (Themes)

Recognizing the Need for Sleep Education	***13 (92.9%)***
"I think so for sure. I'd actually never thought about it until this, but definitely now that I'm thinking about it, I definitely think that it would be beneficial." (Grade 1 Teacher)<br>"I would say yes. I think it's really important that they understand what is happening to your body when you get good sleep and what happens when you don't get good sleep. And that might help them to better their sleep habits" (Grade 4/5 Teacher)	
Lack of Sleep Resources in the Classroom	***12 (85.7%)***
"No, I don't use resources. I haven't kind of stumbled upon any and there's never been any PD. If you asked any teacher in my school, we talked about kids who are tired all the time, but we've never talked about sleep resources. Nobody's ever brought up sleep or sleep education." (Grade 3 Teacher)<br>"No, that is the because the curriculum right now is just one outcome and it's saying like discuss the impacts of sleep and it has no direction on it. There's nothing out there that is available to teachers unless they Google stuff." (Grade 6 Teacher)	
Informal Conversations	***12 (85.7%)***
"I'd have one-on-one conversations with them about like how late were you up last night? Why weren't you going to bed a little bit earlier? How early did you get up and why didn't go back to sleep? Not usually as a whole group." (Grade 1 Teacher)<br>"We talk a little bit about the importance of if something is going on, like if you didn't sleep well or you're tired. We have informal conversations about it. But I don't teach it explicitly." (Grade 6 Teacher)	
Existing Sleep-Related Classroom Strategies	***9 (64.3%)***
"I have done a mindfulness activity. I've used the Calm app" (Grade 4/5 Teacher)<br>"I do use some social skill lessons. I use social stories quite a bit as well." (Grade 6 Teacher)	
Reinforcing Sleep Education at Home	***9 (64.3%)***
"I also think there's a need for parent education. It's all fine to teach the kids, but if there's nobody to back it up at home, then that's a big one. I think it needs to definitely be a partnership between home and school." (Grade 6 Teacher)<br>"Ultimately sleep is controlled by their parents. Continue the conversation at home with their parents." (Grade 3 Teacher)	

Supplementary Table S5. Teachers' Thoughts on Program Development (Themes Organized by User Experience Domains)

**Credible**	
Evidence-Based Program	***14 (100%)***
"If I had to pick between a sleep book written by people who've been doing the research or just a sleep book written by people who wanted to write a nice story about sleeping, I would pick the research based 110 times." (Grade 6 Teacher)<br>"Knowing that there's research behind it would give me more confidence with parents, and more confidence just delivering it and with my administrators, too" (Grade Primary Teacher)	
**Accessible**	
Enhance Accessibility for Families	***10 (71.4%)***
"I would really like to see resources for parents, pre-made resources for parents. I'd send it home and especially if there was a piece in there for parents, this is how many hours of sleep your kids should be getting, that would be great or a letter to parents." (Grade 6 Teacher)<br>"Something translatable for parents or more in parent-friendly language. I think that if the parents could read that book to their kids, that would also be awesome. Having it accessible to parents, it only goes so far if their families aren't on board." (Grade 1 Teacher)	
Language Accessibility	***6 (42.9%)***
"Just to note that you know a huge portion of the population in Nova Scotia, it does their instruction in French." (Grade 2 Teacher)<br>"I have a lot of families that don't speak English. It being translatable would be beneficial" (Grade 1 Teacher)	
**Desirable**	
Appeal of the Storybook	***14 (100%)***
"I really loved it. It would be perfect and approachable." (Grade 1 Teacher)<br>"That was so cute. I really liked like how it worked through. I really liked all the letters. It was sweet" (Grade 6 Teacher)	
Illustration Recommendations	***7 (50%)***
"I think they would really like to see…more illustrations…that might match the themes scenarios (e.g., where it talks about not having caffeine before bed, having a picture that matches that)." (Grade 4/5 Teacher)	

"….for younger students, the visuals are great. But, if you were using it for Grades 6 to 8, you would need differen visuals." (Grade Primary Teacher)	
Diversity Recommendation	***3 (21.4%)***
"More diversity in of the characters. There were some characters in there like characters of colour, but I think I would have liked to see a little bit more." (Grade 4/5 Teacher)<br>" I look for diversity in the characters I teach… and I saw maybe one or two." (Grade ½ Teacher)	
**Valuableness**	
Electronics and Sleep	***14 (100%)***
"I love that it touched on electronics because students mention 'I stole my mom's phone in the middle of the night' or 'I was playing games all night'" (Grade 1 Teacher)<br>"I think it still has great information like talking about the electronics component, that they should have it turned off an hour before. I think that would be that's great for them because a lot of them do stay up late and they're staying on their electronics, so having that alternative and having that suggestion there is good for them". (Grade 4/5 Teacher)	
Sleep Environment and Routine	***7 (50%)***
"I liked that it talked about being in the same environment every night and then going to bed at the same time every night. Because when I am talking to certain students, it's the problems are often 'oh well, I fell asleep on the couch'" (Grade 1 Teacher)<br>"Everything that was in there was still relevant to them, especially thinking back to the schedule part about it being consistent, not being different on the weekends than on weekdays" (Grade 6 Teacher)	
Nutrition and Exercise	***6 (42.9%)***
"The need to stopping eating and to not eat sugar and all of that close to bedtime is important." (Grade 6 Teacher)<br>"Watching what they eat, especially things with caffeine is good information" (Grade 6 Teacher).	
**Useful**	
Incorporate Discussion Points into Program	***14 (100%)***
"I think discussion points would be great or like questions that you could ask whether it's older students versus younger students. I think that would be an awesome idea." (Grade 1 Teacher)<br>"Yes, I think that's a great idea. Little discussion points with the kids. You could ask kids to prompt further engagement too. Ready to go bullet points" (Grade Primary Teacher)	
Incorporate Activity Book into Program	***13 (92.9%)***

"If there was a little activity book. Little colouring pages that they could take home and then talk to their parents about. If it was well targeted to the points in the book I think it could be helpful" (Grade Primary Teacher)<br>"I think if it was a collection of possible activities, pick and choose what works for your class or within your class. If it was like a pdf with a bunch of different activities in it where we could just print the pages that were relevant, then that would be helpful." (Grade 6 Teacher)	
Incorporate Parent Involvement	***10 (71.4%)***
"Discussion questions and questions that I could easily pass on to the parents as well. You can also have these same discussions at home with your children. I would love for it not just for be for students, but for the parents as well because I think it's going to need to be reinforced at home." (Grade 6 Teacher)<br>"I was going to let parents know we're talking about sleep and we have this lovely read-aloud at home, each child's going to get a turn to take it home for a week and share it with you at home" (Grade Primary Teacher)	
Incorporate Activities Into Program	***10 (71.4%)***
"I liked like going back again to the ABC part out of it. Like that alphabetical component, an activity touching on each individual letter, and the kids could come up with brainstorm ideas for what that would look like" (Grade 4/5 Teacher)<br>"An activity that had them reflecting on how they go to bed every night and how something they could do better" (Grade 1 Teacher)	
Incorporate Connections to the Real World in the Program	***6 (42.9%)***
"Allow something that would allow them to make connections so that they could relate to it more." (Grade 4/5 Teacher)<br>"Something where they can see it happen in a story really helps them put themselves in it and it gets to my kids who might not be able to make those connections on their own." (Primary Teacher)	
Culturally Sensitive Information	***6 (42.9%)***
"It's possible that some of the students in my classroom, they don't have that opportunity to have a quiet room. Maybe they don't have a room for themselves and they have to share a room with a sibling. So that might be difficult, that they don't connect" (Grade 4/5 Teacher)	

"Some factors that I've been made aware of to do with this disruption have to do with housing insecurity. You know, not having a home. So sleeping in different people's homes. A lot of siblings in a certain room" (Grade Primary Teacher)	
Incorporate Program into Classroom Lessons	***5 (35.7%)***
"Have a mini-lesson of like what are some things that you do to go to sleep. A mini-lesson that talks about like bedtime routine" Grade 4/5 Teacher)<br>"I think it would have to go along with like a mini-lesson	

Supplementary Table S6. Teachers' Thoughts on Program Delivery (Themes organized by User Experience Domains)

**Findable**	
Benefits of Having a Physical Copy	***13 (92.9%)***
"I think a hard copy would be strongly preferred just because there are a lot of families that are worried about the screen time at school." (Grade Primary Teacher)<br>"I still hold up the book and show the page. A lot of students they like to be able to put their hands on the book." (Grade 2 Teacher)	
Benefits of Having a Digital Copy	***10 (71.4%)***
"I really enjoy playing read-aloud with my class because it makes it so you are able to play it on a big screen and they're all able to see it." (Grade 4/5 Teacher)<br>"Having a digital for like reading as a full class is really nice." (Grade 6 Teacher)	
**Usable**	
Program Delivery in the Curriculum	***14 (100%)***
"Yeah, definitely. I think all like all of it would be relevant for the curriculum for sure, there's always a way to tie it all in." (Grade 4/5 Teacher)<br>"Yes, absolutely. We talk a lot about healthy lifestyles. We talk about exercise a lot.<br>We talk about active and passive hobbies. I think that the way the book is right now would work just fine with the curriculum" (Grade 2 Teacher)	
Ways Teachers Discover the Program	***14 (100%)***
"Normally we would hear from our administration, so our admin sends us different links or emails with different suggestions for things that we might be interested in. Or it would be word of mouth. A lot of teachers at my school, they are great with looking into new programs or signing up for different programs so definitely. I would hear from some of them." (Grade 4/5 Teacher)<br>"Usually that something might come from my principal. Another possibility is through the Nova Scotia Teachers Union which has conferences for each particular association" (Grade 2 Teacher)	
Online Learning Module for Teachers	***13 (92.9%)***
"Yes, as long as it's not too long. Teachers would participate in it." (Grade 6 Teacher)<br>"Absolutely. Absolutely yes. If they had some background knowledge available through a module, I think that would be very helpful" (Grade 2 Teacher)	
Additional Teaching Resources	***13 (92.9%)***

“Having something that you can reference like a Google document that you can reflect back on when you're teaching it years down the road.” (Grade 1 Teacher)	
“I think having some lesson plans available for teachers to go with the book, and if there are like things that need to be photocopied like to have that with the book and some ready-to-go lessons for you, some access to some background information would be very helpful” (Grade 2 Teacher)	
Length of Time Book Would be Used	***12 (85.7%)***
“Depending on the student, some students every day, other students, it might be once a week” (Grade 6 Teacher)	
“I usually do one thing and then I move on. So you know, but I could see that you know, coming up in multiple years.” (Grade 2 Teacher)	
Optimal Timing for Storybook Use	***8 (57.1%)***
“Read the story to the kids when it's silent reading time or independent reading time, or if it's like reading buddies” (Grade 3 Teacher)	
“I would build it right into their schedule, that there would be time to review that each day.” (Grade 6 Teacher)	

Supplementary Table S7. Teachers' Needs Based on Lower and Upper Elementary Students Themes

Grade Level of Storybook	***14 (100%)***
"I think it would be really good up until grade four and then after that would just depend on the teacher and the class on whether they think it would be too young." (Grade 6 Teacher)<br>"It seems like it would be good for the Primary. 4 to 8-year-olds, you could go a little higher too and probably read it to 9 to 10-year-olds as well" (Grade Primary Teacher)	
Vocabulary Level	***13 (92.9%)***
"I think it had some relevant language for them because I always want to push them to expand their vocabulary and I think that would work and I liked the alphabet component where it's spelling out the word sleeping." (Grade 4/5 Teacher)<br>"All language that they would understand, I wouldn't have to do a lot of front loading with them so. I definitely think I could use it with them." (Grade 6 Teacher)	
Age-Level Content Recommendations	***12 (85.7%)***
"I think for Grade 6 it would have to be at a bit of a higher level. If there were additional like pieces to maybe go with it, pulling extra pieces out and for the actual talking about sleep." (Grade 6 Teacher)<br>"It would have to somehow be upgraded a bit. The pictures would have to change a bit, but for Primary to three or P to 3-4, yeah it would work great. For grades 4, 5, and 6 you need to change the look of the illustrations a bit. The words are fine because you've got some big words in there and big concepts." (Grade 3 Teacher)	
